# A beam model and Boltzmann solver for radiotherapy treatment planning of superficial brain metastases using a scanned electron beam at ultra-high (FLASH) dose rate

**DOI:** 10.1088/1361-6560/ae6225

**Published:** 2026-05-06

**Authors:** J Bedford, M Gross, F Riemer, Z Amirkhanyan, F Stephan, U Oelfke

**Affiliations:** 1 Joint Department of Physics, The Institute of Cancer Research and The Royal Marsden NHS Foundation Trust, London SM2 5PT, United Kingdom; 2 Deutsches Elektronen-Synchrotron (DESY), Platanenallee 6, 15738 Zeuthen, Germany

**Keywords:** electron beam, pencil-beam scanning, proton therapy, Boltzmann solver, FLASH, biological effect

## Abstract

*Objective.* Contemporary particle accelerators allow for the generation of a narrow pencil beam of electrons which can be scanned to deliver a clinical dose distribution at an ultra-high (FLASH) dose rate. This study develops a radiotherapy beam model and discrete ordinates Boltzmann solver for such an accelerator and then applies the method to treatment planning for superficial brain metastases. *Approach.* Beam profiles for the quasi-monoenergetic 17.5 MeV electron beam from the Photo Injector Test facility at Deutsches Elektronen-Synchrotron laboratory in Zeuthen (PITZ) were measured at various depths in a water tank using radiochromic film. The incident radiation was modelled as a Gaussian source and the electron distribution in the patient was modelled using classical observations with continuous slowing down approximation (CSDA). This distribution then formed the fixed source component in a discrete ordinates Boltzmann solver. The dose calculation method was then applied to a retrospective study of six patients with superficial brain metastases. The dose due to scanned electrons was compared with that from a single passively scattered proton beam at ultra-high dose rate (UHDR), a proton arc, and a robotic photon treatment. *Main results.* The calculated dose distribution in a homogeneous water phantom agreed with the measured data to within the 3% experimental uncertainty at all depths. Scanned electron beams were able to provide dose distributions for superficial brain metastases that had a better conformity index than either passively scattered protons or robotic photon treatment (1.02 ± 0.13 versus 1.54 ± 0.13 and 1.35 ± 0.26 respectively; median ± hemi-range; p < 0.05). Brain V_12Gy_ and skin dose were acceptable for all treatments. *Significance.* The dose calculation provides a fast and efficient method for inverse planning in the potential clinical application of a scanned electron beam at UHDR. The results show that such an approach could be useful in the treatment of superficial target volumes.

## 1. Introduction

There is currently considerable interest in radiotherapy delivered at ultra-high dose rate (UHDR), where reduced impact on normal tissues has been observed (Dewey and Boag 1959, Bedford and Mitchell 1973, Favaudon *et al* 2014, Diffenderfer *et al* 2020). Many studies aim to understand and maximise this so-called FLASH effect (Diffenderfer *et al* 2022, Favaudon *et al* 2022, Vozenin *et al* 2022).

Several machines have been able to deliver practical treatments at the 100 Gy s^−1^ dose rate that is necessary for such an effect to be observed (Bourhis *et al* 2019a, Mascia *et al* 2023). Such developments are often made using modified clinical platforms where the modifications fundamentally affect the delivery mode and safety framework of the machine, indicating the need for careful consideration of technical standards (Pensavalle *et al* 2024). However, the largest dose rates are produced by particle accelerators in various physics laboratories around the world (Esplen *et al* 2020). One such accelerator is the electron accelerator at the Photo Injector Test facility in Zeuthen (PITZ), part of the Deutsches Elektronen-Synchrotron laboratory (DESY) (Stephan *et al* 2022). This can produce an average dose rate in the order of 10^9^ Gy s^−1^ and is therefore of interest in determining the magnitude of the FLASH effect at dose rates well beyond that of current clinical machines. Practical application of the beam is assisted by beam deflection methods which steer the beam in a scanned delivery pattern, so that almost arbitrary transverse dose distributions can be irradiated within a total treatment time of one millisecond. It is therefore valuable to investigate what might be clinically achievable with the beam at its current energy and this is the goal of the present study.

For calculation of dose from electron beams, several Monte Carlo simulation packages are available and in many cases are in clinical use (Baró *et al* 1995, Agostinelli *et al* 2003, Kawrakow *et al* 2006, Perl *et al* 2012, Battistoni *et al* 2016). However, a discrete ordinates Boltzmann solver has recently been developed to enable fast and accurate inverse planning in radiotherapy (Bedford 2019, 2023). This method can be applied throughout the inverse planning process (Bedford 2024) and it is consequently a valuable approach for use in the investigation of scanned electron beams. The main goal of this strategy is to obtain dose calculation accuracy comparable to Monte Carlo simulation but in a much shorter timeframe, and in a form that can be embedded conveniently into the inverse planning process itself rather than merely as a final dose calculation. This study therefore aims to develop a beam model for the scanned electron beam at PITZ and to calculate dose using the discrete ordinates method. Electron transport is highly dominated by multiple small-angle scattering (Goudsmit and Saunderson 1940a, 1940b), and this is challenging for the discrete particle directions used in the discrete ordinates method, so the approach taken in this study is to use a classical fluence model for calculation of fixed sources, and then to use this as the foundation for the Boltzmann solver. The classical fluence model also serves to stabilise the Boltzmann solver in the same way that exponential primary fluence acts in a photon solver.

Potential clinical applications for the electron beam at its current energy are limited, due to the limited range of electrons at 17.5 MeV, but treatment of superficial brain metastases is one important application. These are typically treated with robotic photon radiotherapy such as Cyberknife (Accuray, Inc., Sunnyvale, CA) or Gamma Knife (Elekta AB, Stockholm, Sweden), where the whole normal brain receives a low dose of radiation due to the passage of the photon irradiation. The present study therefore compares the scanned electron beam including the FLASH effect that is expected, with Cyberknife treatment, and also with proton therapy, which is currently the predominant method for clinical delivery of radiotherapy with UHDRs (Bourhis *et al* 2019b) and which has also been occasionally used for treatment of brain metastases (Atkins *et al* 2018).

## 2. Materials and methods

### 2.1. Beam characteristics and measurements

The key characteristics of the electron beam used in this work are given in table 1. The beam was unfocussed and delivered through a titanium exit window of 50 *µ*m thickness. A plotting tank with 10 mm thick PMMA walls was positioned 26 mm away from the exit window. Due to the horizontal beam line, the beam was delivered through the side wall of the plotting tank, but in modelling the beam, the side wall was considered to be radiologically equivalent to 12 mm of water, following standard clinical practice for the superficial region of electron beams (Khan *et al* 1991). Gafchromic EBT films (Ashland Inc., Wilmington, DE) were used for measurement of beam profiles, the first located at 10.85 mm beyond the wall of the plotting tank, and the remaining located at 11.85 mm intervals. Before modelling, the profiles were symmetrised by averaging the two sides together.

**Table 1.** Key parameters for the electron beam used in this study.^a^

Parameter	Value for pencil beam scanning
Peak energy (MeV)	17.5
Energy full width at half maximum (MeV)	0.3
Beam *x*-standard deviation (mm)^a^	3.8
Beam *y*-standard deviation (mm)^a^	5.4
Bunch length (ps)^b^	30
Bunch interval (*µ*s)^b^	1.0
Bunches per train^b^	1000
Train interval (ms)	99.0

^a^ Note that the beam sizes of the individual electron bunches used for pencil beam scanning can also be reduced by about a factor of 10 by focussing the electron beam before scanning it. A corresponding optimisation could be the topic of future activities.

^b^ Electron bunches were individual bunches in the electron bunch train.

The electron beam was modelled as a quasi-parallel beam of source-axis distance of 10 m. The beam was taken to have Gaussian profiles of 3.8 mm and 5.4 mm standard deviation in the two orthogonal directions, respectively. The beam energy spectrum was also modelled as a Gaussian distribution with peak energy at 17.5 MeV and FWHM 0.3 MeV, truncated below 17.0 MeV and above 18.0 MeV.

### 2.2. Discrete ordinates Boltzmann solver

The beam was transported into the patient model using ray-tracing, with energy loss calculated by CSDA:

\begin{equation*}E\left( {{r_1}} \right) = E\left( {{r_0}} \right) - \left( {{r_1} - {r_0}} \right)S\left( {{r_0}} \right)\frac{{\rho \left( {{r_0}} \right)}}{{{\rho _{\mathrm{e}}}\left( {{r_0}} \right)}}\end{equation*}

where *r*_0_ was the initial radiological path length of the electrons, *r*_1_ was the final radiological path length, $E\left( {{r_0}} \right)$ was the initial energy of the electron fluence, $E\left( {{r_1}} \right)$ was the final energy of the electron fluence, $S\left( {{r_0}} \right)$ was the mass stopping power of the electrons, while $\rho \left( {{r_0}} \right)$ and ${\rho _{\mathrm{e}}}\left( {{r_0}} \right)$ were the mass density and relative electron density of the material, respectively. The appearance of the relative electron density in this equation was to convert from radiological path length to physical path length.

The broadening of the electron beam from multiple small-angle scattering was modelled using the mass scattering power, ${T \mathord{\left/ {\vphantom {T \rho }} \right. } \rho }$, as defined in ICRU 35 (1984). This was the increase in mean square angle of scattering, $\overline {{\theta ^2}} $, per unit of radiological path length, $\rho \;\operatorname{d} l$:

\begin{equation*}\frac{T}{\rho } = \frac{1}{\rho }\,\frac{{{\mathrm{d}}\overline {{\theta ^2}} }}{{{\mathrm{d}}l}}.\end{equation*}

This was calculated using equation (2.8) of International Commission on Radiation Units and Measurements (1984):

\begin{equation*}\frac{T}{\rho } = \pi {\left( {\frac{{2{r_{\mathrm{e}}}Z}}{{\left( {\tau + 1} \right){\beta ^2}}}} \right)^2}\frac{{{N_{\mathrm{A}}}}}{{{M_{\mathrm{A}}}}}\left\{ {\ln \left[ {1 + {{\left( {{\theta _{\mathrm{m}}}/{\theta _\mu }} \right)}^2}} \right] - 1 + {{\left[ {1 + {{\left( {{\theta _{\mathrm{m}}}/{\theta _\mu }} \right)}^2}} \right]}^{ - 1}}} \right\}\,\end{equation*}

where *r*_e_ was electronic radius, *Z* was atomic number and *τ* was the ratio of kinetic energy, *E*, of the electrons to rest energy. *E* was calculated in MeV, so that $\tau = {E \mathord{\left/ {\vphantom {E {0.511}}} \right. } {0.511}}$ for practical purposes. The factor *β* was the ratio of velocity of the electrons to the velocity of light in vacuum and could be calculated in practice from the relationship ${\beta ^2} = {{\tau \left( {\tau + 2} \right)} \mathord{\left/ {\vphantom {{\tau \left( {\tau + 2} \right)} {{{\left( {\tau + 1} \right)}^2}}}} \right. } {{{\left( {\tau + 1} \right)}^2}}}$. *N*_A_ was Avogadro’s number and *M*_A_ was the molar mass of the scattering material.

The angles *θ*_m_ and *θ_µ_* related to the screening effect of orbital electrons around the nucleus, *θ*_m_ being the cutoff angle due to the finite size of the nucleus and *θ_µ_* being the screening angle for small deflections (Rossi 1952). These factors were calculated as:

\begin{equation*}{\theta _{\mathrm{m}}} = \frac{{2{A^{ - 1/3}}}}{{\alpha \beta \left( {\tau + 1} \right)}}\end{equation*}

and:

\begin{equation*}{\theta _\mu } = 1.130\frac{{\alpha {Z^{{1 \mathord{\left/ {\vphantom {1 3}} \right. } 3}}}}}{{\beta \left( {\tau + 1} \right)}}\end{equation*}

where *A* was the nucleon number and *α* was the fine structure constant, taken as ${1 \mathord{\left/ {\vphantom {1 {137}}} \right. } {137}}$.

The mean square angular spread of the Gaussian beam was then taken to be:

\begin{equation*}\overline {{\theta ^2}} \left( z \right) = \overline {\theta _i^2} + \int\limits_0^z T\left( u \right){\mathrm{d}}u\end{equation*}

where the initial mean square angular spread, $\overline {\theta _{\mathrm{i}}^2} $, was taken to be zero due to the parallel nature of the incident beam. The angular distribution of fluence, ${\phi _\theta }\left( {\theta ,z} \right)$, was then calculated from the normal distribution:

\begin{equation*}{\phi _\theta }\left( {\theta ,z} \right) = \frac{1}{{\cos \theta }}\;\frac{1}{{\pi \overline {{\theta ^2}} \left( z \right)}}\exp \left( {\frac{{ - {\theta ^2}}}{{\overline {{\theta ^2}} \left( z \right)}}} \right)\end{equation*}

where the ${1 \mathord{\left/ {\vphantom {1 {\cos \theta }}} \right. } {\cos \theta }}$ factor was a correction from planar fluence to scalar fluence (and where it should be noted that the variance of a Gaussian distribution is equal to half of the mean square). The mean square radial spread (i.e. the spread in beam width as opposed to the spread in beam direction), $\overline {{r^2}} $, was calculated as:

\begin{equation*} \overline{r^2}\left(z\right) = \overline{r_i^2} + \int_0^z \left(z - u\right)^2 T\left(u\right) \, \mathrm{d}u \end{equation*}

where $\overline {r_{\mathrm{i}}^2} $ was the initial lateral mean square spread of the incident fluence. The lateral distribution of fluence was then calculated as a Gaussian distribution of the form:

\begin{equation*}{\phi _{\mathrm{r}}}\left( {r,z} \right) = \frac{1}{{\pi \overline {{r^2}} \left( z \right)}}\exp \left( {\frac{{ - {r^2}}}{{\overline {{r^2}} \left( z \right)}}} \right).\end{equation*}

Computationally, this was accomplished by starting with a delta function, convolving it with a series of Gaussian functions of progressively broadening mean square, and then looking up the fluence distribution of appropriate width at each depth in the patient when ray tracing.

The scalar fluence, *φ*, was calculated from an empirical relationship based on equation 2.44 of International Commission on Radiation Units and Measurements (1984) as:

\begin{equation*}{\phi _z}\left( z \right) = \exp \left( { - {{\left( {1 - 1/b} \right)}^{1 - b}}{{\left( {z/{R_{{\mathrm{ex}}}}} \right)}^b}} \right)\left( {1 + c{z^{1/b}}} \right)\end{equation*}

where *b* represented the steepness of the fluence reduction with depth and *c* represented the magnitude of the initial buildup of scattered fluence. *R*_ex_ was the extrapolated practical range of the beam, which was taken to be $2.825\left( {1.0 + 0.0714\sqrt S } \right)$ times the energy, in MeV, of the fluence, where *S* was the equivalent square field size, in mm. The two terms in the right-hand bracket represented the transmission of unscattered fluence, and the production of scattered fluence, respectively. The parameters *b* and *c* were set empirically to 2.0 and 0.1, respectively, and radiological path length was used in the evaluation of this formula.

The distribution of classical fluence was thus calculated as:

\begin{equation*}\phi \left( {r,\theta ,z} \right) = {\phi _{\mathrm{r}}}\left( {r,z} \right)\,{\phi _\theta }\left( {\theta ,z} \right)\,{\phi _{\mathrm{z}}}\left( z \right)\,.\end{equation*}

This fluence, due to multiple small-angle scattering, was treated as a fixed source in the Boltzmann solver, which was used to include large-angle scattering. The solver itself was identical to that described previously (Bedford 2024) so is only briefly summarised here. The angular quadrature was a simple scheme based on IEC 60601 gantry and couch angles, with ordinates spaced at intervals of 30° of gantry and couch angle. Energy ordinates were from 1 to 18 MeV at intervals of 1 MeV. The equations to be solved were (Hensel *et al* 2006):

\begin{align*} {{\boldsymbol{\Omega }}_{\operatorname{e}} } \cdot \nabla {\varPhi _{\operatorname{e}} }\left( {{\mathbf{r}},{{\boldsymbol{\Omega }}_{\operatorname{e}} },{E_{\operatorname{e}} }} \right)&amp; = {\rho _{\mathrm{e}}}\left( {\mathbf{r}} \right)\int\limits_0^\infty {\int\limits_{4\pi } {{{\tilde \sigma }_{\operatorname{M}} }\left( {{{E^{\prime}}_{\mathrm{e}}},{E_{\mathrm{e}}},{{{\boldsymbol{\Omega ^{\prime}}}}_{\mathrm{e}}} \cdot {{\boldsymbol{\Omega }}_{\mathrm{e}}}} \right){\varPhi _{\mathrm{e}}}\left( {{\mathbf{r}},{{{\boldsymbol{\Omega ^{\prime}}}}_{\mathrm{e}}},{{E^{\prime}}_{\mathrm{e}}}} \right)d{{{\boldsymbol{\Omega ^{\prime}}}}_{\mathrm{e}}}d{{E^{\prime}}_{\mathrm{e}}}} } \nonumber\\ &amp; \quad + {\rho _{\operatorname{c}} }\left( {\mathbf{r}} \right)\int\limits_{4\pi } {{{\tilde \sigma }_{\operatorname{Mott} }}\left( {{\mathbf{r}},{E_{\operatorname{e}} },{{{\boldsymbol{\Omega ^{\prime}}}}_{\operatorname{e}} } \cdot {{\boldsymbol{\Omega }}_{\operatorname{e}} }} \right){\varPhi _{\operatorname{e}} }\left( {{\mathbf{r}},{{{\boldsymbol{\Omega ^{\prime}}}}_{\operatorname{e}} },{E_{\operatorname{e}} }} \right)d{{{\boldsymbol{\Omega ^{\prime}}}}_{\operatorname{e}} }} \nonumber\\ &amp; \quad - {\rho _{\operatorname{e}} }\left( {\mathbf{r}} \right)\sigma _{\operatorname{M}} ^{\operatorname{tot} }\left( {{E_{\operatorname{e}} }} \right){\varPhi _{\operatorname{e}} }\left( {{\mathbf{r}},{{\boldsymbol{\Omega }}_{\operatorname{e}} },{E_{\operatorname{e}} }} \right) - {\rho _{\operatorname{c}} }\left( {\mathbf{r}} \right)\sigma _{\operatorname{Mott} }^{\operatorname{tot} }\left( {{\mathbf{r}},{E_{\operatorname{e}} }} \right){\varPhi _{\operatorname{e}} }\left( {{\mathbf{r}},{{\boldsymbol{\Omega }}_{\operatorname{e}} },{E_{\operatorname{e}} }} \right)\end{align*}

where ${\varPhi _{\mathrm{e}}}$ denoted electron fluence, ${{\boldsymbol{\Omega }}_{\mathrm{e}}}$ was a unit normal in the direction of interest, **r** was the position of interest and ${E_{\mathrm{e}}}$ was the electron energy of interest. During interactions, initial direction and energy were given by ${{\boldsymbol{\Omega ^{\prime}}}_{\mathrm{e}}}$ and ${E^\prime_{\mathrm{e}}}$, respectively, and final direction and energy were given by ${{\boldsymbol{\Omega }}_{\mathrm{e}}}$ and ${E_{\mathrm{e}}}$, respectively. ${\rho _{\mathrm{e}}}\left( {\mathbf{r}} \right)$ was the electron density at position **r**, while ${\rho _{\mathrm{c}}}\left( {\mathbf{r}} \right)$ was the density of atomic cores. ${\tilde \sigma _{\operatorname{M}} }\left( {{{E^{\prime}}_{\mathrm{e}}},{E_{\mathrm{e}}},{{{\boldsymbol{\Omega ^{\prime}}}}_{\mathrm{e}}} \cdot {{\boldsymbol{\Omega }}_{\mathrm{e}}}} \right)$ was the Møller cross section differential in energy and angle and ${\tilde \sigma _{\operatorname{Mott} }}\left( {{\mathbf{r}},{E_{\operatorname{e}} },{{{\boldsymbol{\Omega ^{\prime}}}}_{\operatorname{e}} } \cdot {{\boldsymbol{\Omega }}_{\operatorname{e}} }} \right)$ was the Mott cross section differential in energy and angle. The corresponding quantities $\sigma _{\operatorname{M}} ^{\operatorname{tot} }\left( {{E_{\operatorname{e}} }} \right)$ and $\sigma _{\operatorname{Mott} }^{\operatorname{tot} }\left( {{\mathbf{r}},{E_{\operatorname{e}} }} \right)$ were the total scattering cross sections. Bremsstrahlung was neglected in this model.

The kinematics of Møller scattering dictated that scattering through an angle from one angle ordinate to another also implied a particular loss of energy, so the double integral was replaced with a discrete summation over the *N* angle ordinates, using the appropriate final energy to determine the initial energy involved. Likewise, with Mott scattering, where there was no energy change during scattering, the integral could be reduced to a summation over angle ordinates. Thus, the large-angle scattering sources could be calculated as:

\begin{align*} Q_{nijk}^{\operatorname{scat} }\left( {x,y,z} \right)&amp; \cong {\rho _{\operatorname{e}} }\left( {\mathbf{r}} \right)\sum\limits_{n = 1}^N {{{\tilde \sigma }_{\operatorname{M}} }\left( {{{E^{\prime}}_{\mathrm{e}}},{E_{\mathrm{e}}},{{{\boldsymbol{\Omega ^{\prime}}}}_{\mathrm{e}}} \cdot {{\boldsymbol{\Omega }}_{\mathrm{e}}}} \right){\varPhi _{\mathrm{e}}}\left( {{\mathbf{r}},{{{\boldsymbol{\Omega ^{\prime}}}}_{\mathrm{e}}},{{E^{\prime}}_{\mathrm{e}}}} \right)} \nonumber\\ &amp; \quad + {\rho _{\mathrm{c}}}\left( {\mathbf{r}} \right)\sum\limits_{n = 1}^N {{{\tilde \sigma }_{\operatorname{Mott} }}\left( {{\mathbf{r}},{E_{\operatorname{e}} },{{{\boldsymbol{\Omega ^{\prime}}}}_{\operatorname{e}} } \cdot {{\boldsymbol{\Omega }}_{\operatorname{e}} }} \right){\varPhi _{\operatorname{e}} }\left( {{\mathbf{r}},{{{\boldsymbol{\Omega ^{\prime}}}}_{\operatorname{e}} },{E_{\operatorname{e}} }} \right)}\end{align*}

where *i, j* and *k* indexed the voxels in the *x, y* and *z* directions, respectively, and *n* indexed the *N* angular ordinates. Equation (12) were then solved using standard transport sweeps (Lewis and Miller 1984):

\begin{equation*}\varPhi _{nijk}^{\operatorname{scat} } = \frac{{\frac{{2{\mu _n}}}{{\Delta {x_i}}}\varPhi _{n,i - 1/2,jk}^{\operatorname{scat} } + \frac{{2{\eta _n}}}{{\Delta {y_j}}}\varPhi _{ni,j - 1/2,k}^{\operatorname{scat} } + \frac{{2{\xi _n}}}{{\Delta {z_k}}}\varPhi _{nij,k - 1/2}^{\operatorname{scat} } + Q_{nijk}^{\operatorname{scat} }}}{{\frac{{2{\mu _n}}}{{\Delta {x_i}}} + \frac{{2{\eta _n}}}{{\Delta {y_j}}} + \frac{{2{\xi _n}}}{{\Delta {z_k}}} + {\rho _{\operatorname{e}} }\left( {x,y,z} \right)\sigma _M^{\operatorname{tot} } + {\rho _{\mathrm{c}}}\left( {x,y,z} \right)\sigma _{Mott}^{\operatorname{tot} }}}\end{equation*}

where *μ_n_, η_n_* and *ξ_n_* were the direction cosines of the discrete ordinates, *n* (Hensel *et al* 2006), and $\Delta {x_i}$, $\Delta {y_j}$ and $\Delta {z_k}$ were the voxel sizes in the three orthogonal directions. As the classical small-angle fluence remained constant, regardless of the scattering events taking place, this fluence was added to the scattered fluence after each iteration of equation (14):

\begin{equation*}{\varPhi _{nijk}} = \varPhi _{nijk}^{{\mathrm{scat}}} + \varPhi _{nijk}^{{\mathrm{classical}}}\end{equation*}

where $\varPhi _{nijk}^{\operatorname{scat} }$ referred to the scattered fluence calculated from equation (14) and $\varPhi _{nijk}^{{\mathrm{classical}}}$ referred to the unscattered fluence from equation (11). Ten iterations of this scheme were used to give total fluence, from which the absorbed dose, $D\left( {\mathbf{r}} \right)$, was calculated by multiplying fluence by mass collision stopping power, ${S_{\mathrm{e}}}\left( {{\mathbf{r}},{E_{\mathrm{e}}}} \right)$:

\begin{equation*}D\left( {\mathbf{r}} \right) = \frac{1}{{\rho \left( {\mathbf{r}} \right)}}\sum\limits_{e = 1}^{{N_{\mathrm{e}}}} {{S_{\mathrm{e}}}\left( {{\mathbf{r}},{E_{\operatorname{e}} }} \right)\sum\limits_{n = 1}^{{N_a}} {{\varPhi _{\operatorname{e}} }\left( {{\mathbf{r}},{{\boldsymbol{\Omega }}_{\operatorname{e}} },{E_{\operatorname{e}} }} \right)} } \end{equation*}

where the outer summation referred to the *N_e_* energy ordinates and the inner sum referred to the *N_a_* angle ordinates.

Monitor units (MUs) were defined in a similar way to proton pencil beam scanning but in a two-dimensional sense due to the limited penetration of the beam: one MU was defined as giving 1 cGy at the depth of maximum dose to a 100 mm × 100 mm area. In other words, the MU was defined as a global normalisation quantity over a reference area, rather than as a local dose indicator for individual pencil beams. With a 4 mm spot spacing, this corresponded to the delivery of 25 × 25 = 625 pencil beams. A consequence of this definition was that if only one pencil beam was delivered, 1 MU delivered 6.25 Gy to that pencil beam.

### 2.3. Comparison with monte carlo simulation

The beam model and dose calculation were compared against the measured data, but as the measurements were obtained in a homogeneous medium, an additional comparison was made with the results of Monte Carlo simulation. The first of the brain patient cases (see section 2.6 below) was recalculated using the PENELOPE Monte Carlo code (Baró *et al* 1995) using the simulation package PRIMO (Sempau *et al* 2011). The spot patterns for the two electron beams used in the plan were used to create custom phase space files located 30 mm proximal to the isocentre. Each phase space had dimensions 50 mm × 50 mm and resolution 0.01 mm, therefore containing 2.5 × 10^7^ particles with varying statistical weights. Bin size was 2 mm width × 2 mm height × 1 mm length. Final comparison was by means of orthogonal dose profiles through the isocentre.

To further verify the performance of the dose calculation in heterogeneous media, the first of the two beams was shifted and rotated so that it formed a direct anterior beam entering through the frontal sinus, where the density varied from air density to bone density in a complex pattern. The comparison with Monte Carlo simulation was repeated.

### 2.4. Biological effect of UHDR

Relative biological effectiveness (RBE) of the beam in normal tissues, the so-called FLASH effect, was calculated according to the model described by Bedford (2025). The time-structure of the accelerator (table 1) was followed (Shen *et al* 2017) and the dose distribution and dose rate distribution from each pulse was accumulated. The RBE was then calculated as:

\begin{equation*} b\left(\mathbf{r}\right) = f\left( d\left(\mathbf{r}\right), \bar{\dot{d}}\left(\mathbf{r}\right) \right) d\left(\mathbf{r}\right) \end{equation*}

where $d\left( {\mathbf{r}} \right)$ represented the dose at a given point in the patient and $\overline{\dot{d}}(\mathbf{r})$ represented the mean dose rate of the delivery, in Gy s^−1^. In the previous work (Bedford 2025), the variation of $f\left( {\mathbf{r}} \right)$ as a function of mean dose rate was taken as a simple ramp function but in this study, due to the much greater range of dose rates encountered in the treated volume, it was modelled as:

\begin{equation*} f_{r}\left( \bar{\dot{d}}\left(\mathbf{r}\right) \right) = \begin{cases} 0 &amp; \text{for } \bar{\dot{d}}\left(\mathbf{r}\right) \leq T_{R} \\ \tanh \left\{ F_{R} \log_{10} \left( \bar{\dot{d}}\left(\mathbf{r}\right) - T_{R} + 1 \right) \right\} &amp; \text{for } \bar{\dot{d}}\left(\mathbf{r}\right) &gt; T_{R} \end{cases} \end{equation*}

where ${F_{R}}$ was a factor reflecting the strength of the dose rate dependence and ${T_{R}}$ was the dose rate threshold for FLASH to occur. Then $d\left( {\mathbf{r}} \right)$ was modelled according to the meta-analysis of animal studies of Böhlen *et al* (2022), but modulated by the dose rate factor, *f*_*r*_, of equation (18):

\begin{equation*} f\left( d\left(\mathbf{r}\right), \bar{\dot{d}}\left(\mathbf{r}\right) \right) = \begin{cases} 1 &amp; \text{for } d\left(\mathbf{r}\right) \leq T_{D} \\ f_{r}\left( \bar{\dot{d}}\left(\mathbf{r}\right) \right) F_{D} \left( \frac{T_{D}}{d\left(\mathbf{r}\right)} - 1 \right) + 1 &amp; \text{for } d\left(\mathbf{r}\right) &gt; T_{D} \end{cases} \end{equation*}

where *F_D_* was a dose response factor and *T_D_* was a dose threshold. Note that when $d\left( {\mathbf{r}} \right)$ and $\overline{\dot{d}}(\mathbf{r})$ were large, the RBE reduced to $1 - {F_{D}}$, in other words the dose response factor *F_D_* governed the magnitude of the reduction in biological effect.

**Figure 1.**
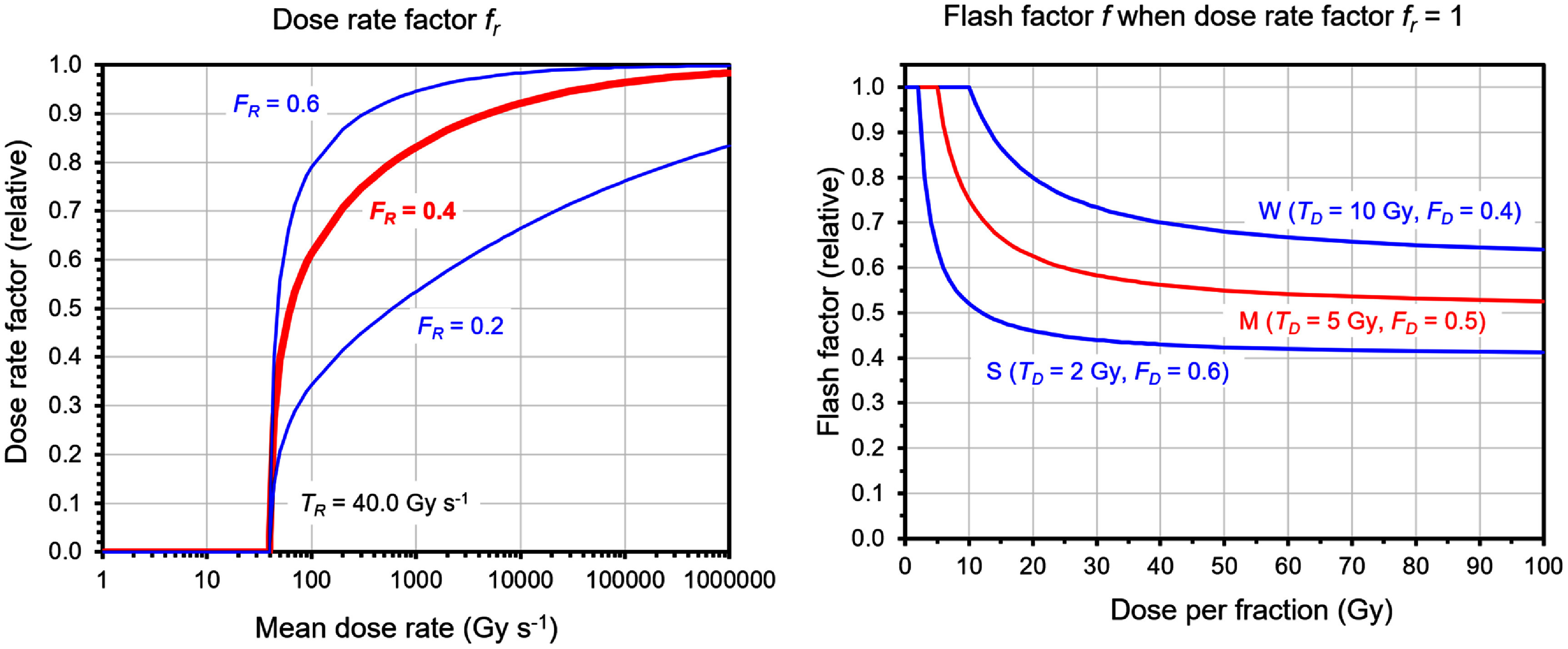
FLASH model used in the study. (a) The dose rate model with dose rate threshold of 40 Gy s^−1^ and several dose rate factors, of which *F_R_* = 0.4 was used throughout the study. (b) The dose model for weak (W), medium (M) and strong (S) FLASH effect, all three of which were considered in the study. Note that (b) shows the dose effect when *f*_*r*_ from (a) equals unity, but the dose effect is typically smaller than this as the value of *f*_*r*_ modulates the magnitude.

Figure 1 illustrates the form of this model and table 2 gives the parameters used for the work in this study. Previous studies indicated that a dose threshold of 10 Gy and sparing of 0.4 were widely observed (Böhlen *et al* 2022), but that FLASH effects at 7 Gy (Montay-Gruel *et al* 2021) and 4 Gy (Chabi *et al* 2021) were also possible, suggesting a much lower dose threshold. Due to this uncertainty, weak, medium and strong FLASH effects were modelled. The weak effect was intended to represent the behaviour described by Böhlen *et al* (2022) and the medium effect was more similar to the studies of Montay-Gruel *et al* (2021) and Chabi *et al* (2021). Moreover, the extremely high dose rate of the electron accelerator was expected (but not proven) to produce a stronger effect, notably with a lower dose threshold and higher dose response factor, and this was modelled by the strong scenario. The choice of the dose rate factor was also intended to allow scope for further FLASH behaviour at extremely high dose rates.

**Table 2.** Parameters used to calculate FLASH effect in weak, medium and strong scenarios.

Parameter	Weak value	Medium value	Strong value
Dose rate threshold, *T_R_* (Gy s^−1^)	40.0	40.0	40.0
Dose rate effect *F_R_* (relative)	0.4	0.4	0.4
Dose threshold, *T_D_* (Gy)	10.0	5.0	2.0
Dose effect, *F_D_* (relative)	0.4	0.5	0.6

The RBE due to FLASH effect was included in all dose calculations during inverse planning, but was set to unity in the gross tumour volume (GTV) itself as this was considered to be comprised of tumour tissue rather than normal tissue.

### 2.5. Inverse planning

The dose calculation algorithm described in section 2.2 was embedded into the inverse planning scheme of in-house software AutoBeam v6.4 (Xing and Chen 1996, Bedford 2013). As the full discrete ordinates calculation was too time-consuming to use for every iteration, a simplified calculation was used at the intermediate steps. This was based on the empirical model of Chen *et al* (2021):

\begin{equation*}D\left( z \right) = \left( {\frac{{{z^{0.1}}}}{{N + {z^{0.2}}}}} \right){e^{ - \mu z}}\left( {1 - \frac{{z - {R_{50}}}}{{\sqrt {{n^{0.5}} + {{\left( {z - {R_{50}}} \right)}^2}} }} + t} \right)\end{equation*}

where *z* was the radiological depth in the patient. In this formula, *N* governed the initial buildup of the beam, *n* governed the slope of the main fall-off of the beam, *µ* (mm^−1^) represented attenuation of Bremsstrahlung and *t* (Gy) represented additional unattenuated Bremsstrahlung. Practically, the value of *N* was taken to be 15.0 and *μ* was taken to be 0.001 mm^−1^. The value of *n* was taken to be 4000.0 *e*, where *e* was the nominal beam energy in MeV, which allowed application of the model to beams of various energy. The value of *R*_50_ was taken as 2.5 *e* and *t* was set to zero. In the lateral direction, the Gaussian incident profile of the beam was used at all depths.

The inverse planning scheme used the biologically effective dose computed in section 2.3, making successive changes to the pencil beam intensities so as to closer match the delivered dose to the desired dose (Xing and Chen 1996, Bedford 2025). The method thus implicitly optimised both dose and dose rate in a manner similar to Gao *et al* (2020).

### 2.6. Patient study

Six patients previously treated with 6 MV photons using Cyberknife were retrospectively considered. All patients had multiple brain metastases resulting from primary tumours elsewhere in the body. In each of the six patients, a single superficial metastasis was selected for study, having a size of greater than 10 mm and a depth of less than 36 mm. Very small volumes were outside of the scope of this study as they required the use of a very narrow electron pencil beam. Moreover, for photons with Cyberknife, this study used a multileaf collimator, whereas for very small volumes, a cone-shaped collimator would be optimal. Deeper volumes were also out of scope due to the limited penetration of the electron beams used in this study. For the six patients, median GTV volume was 1.4 cm^3^ (range 0.5 cm^3^–2.5 cm^3^). Actual prescribed doses varied slightly from 20 to 24 Gy in a single fraction, depending on the primary tumour and the size of the GTV, but for uniformity of comparison, all GTVs were prescribed in this study to 21 Gy in a single fraction to 98% of the GTV. In accord with normal practice in stereotactic radiosurgery, no clinical target margin or planning target margin was used and dose maximum was considered acceptable at up to 42 Gy.

The principal interest in the planning comparison was the volume of normal brain irradiated to 12 Gy. Normal brain was defined as brain with GTV subtracted, and an additional volume of proximal brain was constructed, consisting of normal brain within 10 mm of the GTV. The relevant dose constraint for normal brain was given by Hanna *et al* (2018) as V_12Gy_ < 10 cm^3^. Skin was also considered important and was contoured as the most superficial 5 mm of the patient volume. Proximal skin was considered to be skin within 20 mm of the GTV. The dose constraints given by Timmerman (2022) for skin were 25.5 Gy to 10 cm^3^ and 27.5 Gy to 0.035 cm^3^.

For each patient, six treatment plans were constructed in AutoBeam v6.4 (table 3). The electron treatment plans used two oblique beams so as to spare the skin as much as possible. Although the treatment planning framework was designed to apply electron beams in multiple energy layers similarly to proton pencil beam scanning, only one energy layer was used to avoid time delays in delivery. The energy for each beam was chosen to be equal to the maximum radiological depth of the GTV multiplied by a range-energy factor of 0.4. For example, to give a maximum depth of 40 mm, the beam was chosen to be 16 MeV. Pencil beam scanning was used with a spot separation of 4 mm. Due to the uncertainty in FLASH effect, weak, medium and strong effects were modelled. The dose including FLASH RBE was calculated for each beam separately and then summed as the interval between delivery of the two beams was expected to be considerably longer than 0.1 s, so that the FLASH effects of the two beams were independent (McKay *et al* 2021).

**Table 3.** Summary of treatment plans.

Plan	Details	FLASH
Electrons W	Two 13–14 MeV beams with pencil beam scanning^a^	W (*T_D_* = 10 Gy, *F_D_* = 0.4)
Electrons M	Two 13–14 MeV beams with pencil beam scanning^a^	M (*T_D_* = 5 Gy, *F_D_* = 0.5)
Electrons S	Two 13–14 MeV beams with pencil beam scanning^a^	S (*T_D_* = 2 Gy, *F_D_* = 0.6)
Protons passive scattering	Single 32–67 MeV proton beam with passive scattering^a^	M (*T_D_* = 5 Gy, *F_D_* = 0.5)
Protons arc	Single 30–72 MeV proton beam with 60° arc^a^	M (*T_D_* = 5 Gy, *F_D_* = 0.5) but dose rate too low
Cyberknife photons	171 fixed 6 MV beams in Cyberknife head path	M (*T_D_* = 5 Gy, *F_D_* = 0.5) but dose rate too low

^a^ Energies shown are the range of values selected using range-energy relationships.

The scanned electron beam was compared with proton radiotherapy as the latter was considered to be among the most conformal treatment options currently available. The proton beam was designed to be as simple as possible to facilitate the FLASH effect. Whereas with the electron beams, significant parts of the superficial tissue were expected to receive 10 Gy per beam, giving FLASH effect, the buildup of the proton beam was expected to result in rather lower dose superficially than the electron beam, so that using two beams would result in less than 10 Gy, below the threshold for FLASH. A single proton beam was therefore used. The beam was modelled as passively scattered, having a custom compensator shaped to the distal edge of the GTV in the classical manner. The beam parameters were based on the 230 MeV beam of a double-scattering system (Ion Beam Applications S.A., Louvain-la-Neuve, Belgium) (Slopsema 2012). This had a single source with a source-axis distance of 2300 mm and width and length both equal to 30.0 mm (Slopsema *et al* 2014). No ridge filter was used. The medium model of FLASH was used as the dose rate was unlikely to reach a sufficiently high level to give rise to the strong effect.

The proton arc was included for comparative purposes. Although the medium FLASH effect was calculated, in practice the delivery time was too long for this to affect the dose distribution. The proton arc was manually designed to cover a 60° arc length over the lesion to be treated. Segments were located at 1° intervals of gantry angle, with segment groups of 10°, so that 10 energy layers were produced in each 10° of arc. Each energy layer used pencil beam scanning with a beam width of standard deviation 4 mm and spot spacing of 4 mm (Lin *et al* 2014). The dose calculation was based on a 230 MeV beam (Farr *et al* 2013) delivered through a dedicated nozzle (Ion Beam Applications S.A.).

The final comparison was with the 6 MV unflattened photon beam of a Cyberknife robotic linear accelerator. This was preferred clinical technique for this type of lesion. The standard head path of the robot was modelled, which consisted of 171 non-coplanar beam directions arranged in a bouquet around the patient’s head. The S7 multileaf collimator was simulated, having 52 pairs of leaves of width 3.85 mm at 800 mm (virtual) source-to-axis distance. In clinical treatment planning for these lesions, the orbits plus a 10 mm margin, and the inferior regions of the patients were designated as critical areas to be avoided by beam entry paths. This facility was not available in AutoBeam, so those regions were constrained to a maximum dose of 1 Gy to give a corresponding effect.

For all techniques, dose was calculated by discrete ordinates on an adaptive Cartesian grid of resolution 1.0 mm × 1.0 mm × 1.0 mm except in regions of low incident fluence, where resolution was 4.0 mm × 4.0 mm × 4.0 mm in the interests of speed and memory resources (Bedford 2023, 2024). During inverse planning for protons, intermediate fast calculations were performed using a simple analytical method (Bortfeld and Schlegel 1996, Bortfeld 1997), while during inverse planning for Cyberknife, a fast convolution method was used for intermediate dose calculations (Bedford 2002). For the proton plans, one MU was defined as giving 1 cGy to a 100 mm × 100 mm × 100 mm volume at depth ranging from 50 mm to 150 mm. The variation of proton stopping power in air with energy was taken into account when computing proton MUs and a proton RBE of 1.1 was used throughout, in addition to the FLASH RBE.

For the electron beam, the temporal characteristics given in table 1 were used. The dose rate within a single 5 nC bunch from the accelerator at PITZ for the beam described in table 1 was 2.0 × 10^12^ Gy s^−1^. Noting from section 2.2 that 1 MU ≡ 6.25 Gy, the instantaneous MU rate was taken to be 3.2 × 10^11^MU s^−1^. After allowing for the 3.0 × 10^−5^ duty cycle of the bunches within a train, this corresponded to a mean dose rate within a single pencil beam of 6.0 × 10^7^ Gy s^−1^.

For passively scattered protons, the temporal beam structure (García Díez *et al* 2023) was loosely based on the compact synchrocyclotron (S2C2) of the Proteus ONE system (Ion Beam Applications, S.A.). The beams consisted of a series of pulses, each of the order of 1 ns long, with a repetition period in the order of 10 ns. Approximately 1000 pulses made a train of length in the order of 10 *µ*s. This process was repeated every 1 ms approximately (Jolly *et al* 2020, Nesteruk and Psoroulas 2021). The instantaneous MU rate within each pulse was taken to be 10^7^ MU s^−1^, corresponding to 100 Gy s^−1^ after allowing for the 10^−3^ duty cycle and 10^−2^ conversion from cGy to Gy. This was an optimistic estimate but was considered to be achievable in principle even if difficult in practice.

Proton arcs and Cyberknife plans were also assigned beam structures and dose rates but the mean dose rates were too low for the dose rate threshold of 40 Gy s^−1^ to be exceeded, so these plans produced a FLASH RBE of 1.0.

All plans used similar objectives and constraints, with minor variations on an individual basis as required (table 4). These were biological objectives and constraints, after allowing for RBE. Final plans were compared using two-tailed paired Wilcoxon signed rank tests. Uncertainties were assessed by setting extreme parameters into the electron plan for patient 1 and recalculating the plan. The lower bound was calculating using a prescribed dose of 20 Gy instead of 21 Gy, i.e. 5% low, to simulate 5% uncertainty in dose calculation. The FLASH parameters were set as for the S plan, i.e. lowered normal tissue biologically effective dose, and additionally the dose rate effect (see figure 1) was set to *F_R_* = 0.6 to simulate even stronger FLASH effect. The upper bound of uncertainty was estimated using 22 Gy prescribed dose, W FLASH parameters and *F_R_* = 0.2. The normal values for this patient were considered to be those for the M plan.

**Table 4.** Typical biological objectives and constraints for the inverse planning procedure.

Structure	Objective/constraints	Importance
GTV	Minimise RMS^a^ variation around 30 Gy	1
GTV-3 mm	Minimise RMS^a^ variation around 40 Gy	100
GTV surface 3 mm	Minimise RMS^a^ variation around 30 Gy	100
Brain-GTV	Volume irradiated to 21 Gy	1
Brain-GTV	Volume irradiated to 12 Gy	1
Brain-GTV	Volume irradiated to 5 Gy	1
Brain-GTV	Volume irradiated to 1 Gy	1
Skin	Volume irradiated to 10 Gy	1
Skin	Maximum dose	1
Eye block	Maximum dose	<1 Gy^b^
Inferior block	Maximum dose	<1 Gy^b^

^a^ RMS: Root-mean-square. ^2^For Cyberknife only.

## 3. Results

### 3.1. Beam model

**Figure 2.**
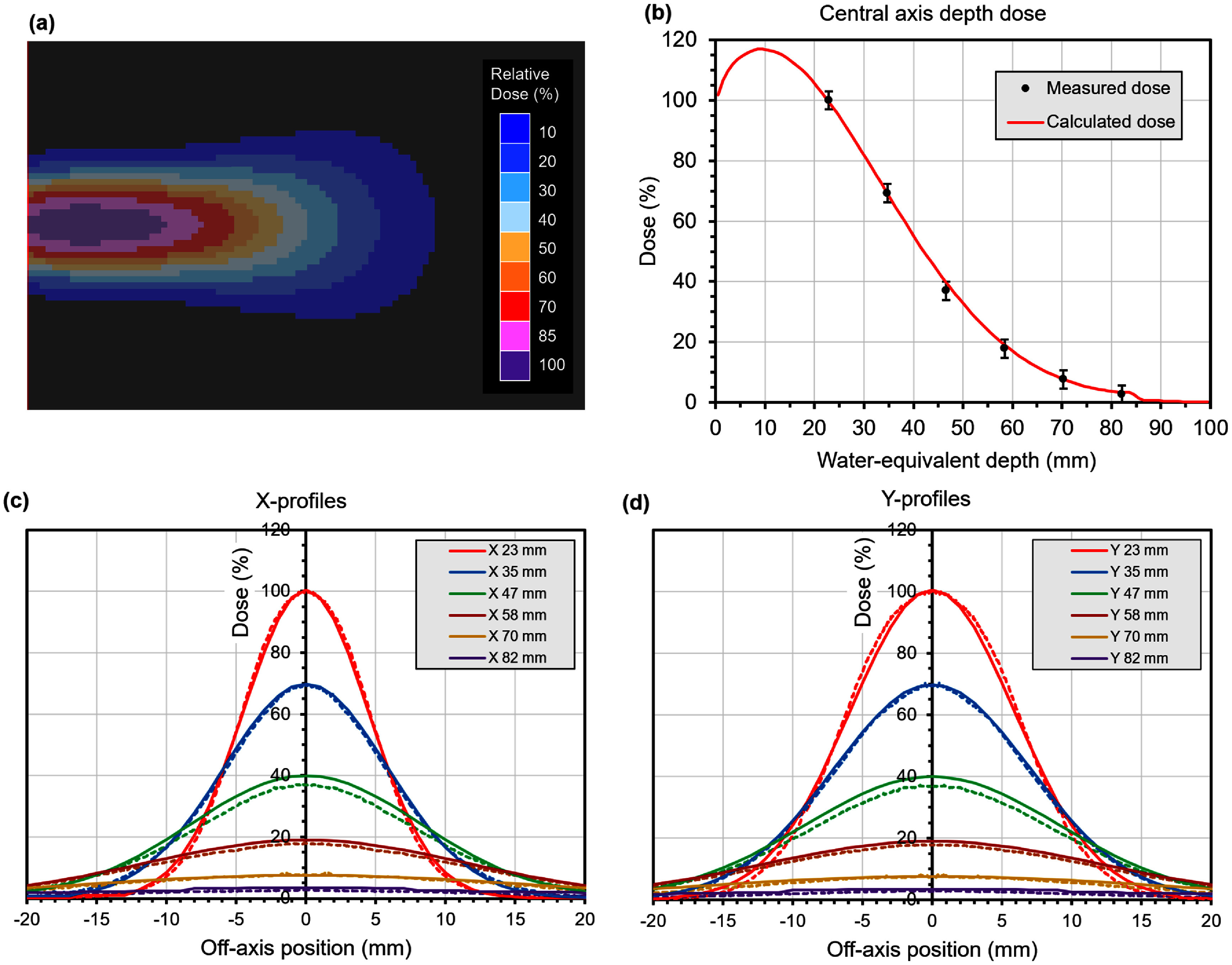
Relative dose in a water phantom from the electron beam. (a) Graphical view of the calculated dose, (b) measured and calculated central axis depth dose curves, (c) measured and calculated *X* beam profiles and (d) measured and calculated *Y* beam profiles. In the profiles, dotted lines are measured dose and solid lines are calculated. Measured doses are symmetrised by averaging left and right measurements together. The error bars on the depth dose curve are for ±3% dose.

The results of modelling the electron beam are shown in figure 2. The measured data are normalised to the most superficial measurement, that at 23 mm radiological depth, and the calculated data are normalised to give agreement with the measured curves as a whole. The central axis depth dose agrees with the measured data to within the experimental uncertainty, with the least good agreement being seen at 47 mm depth, where the difference between measurements and calculations is 3% (100% being defined as the dose at 23 mm depth). The Gaussian forms of the profiles show good agreement between measured and calculated dose. The differences in the central axis depth dose are also included in the profiles, so the profile at 47 mm depth shows some disagreement.

### 3.2. Comparison with Monte Carlo simulation

**Figure 3.**
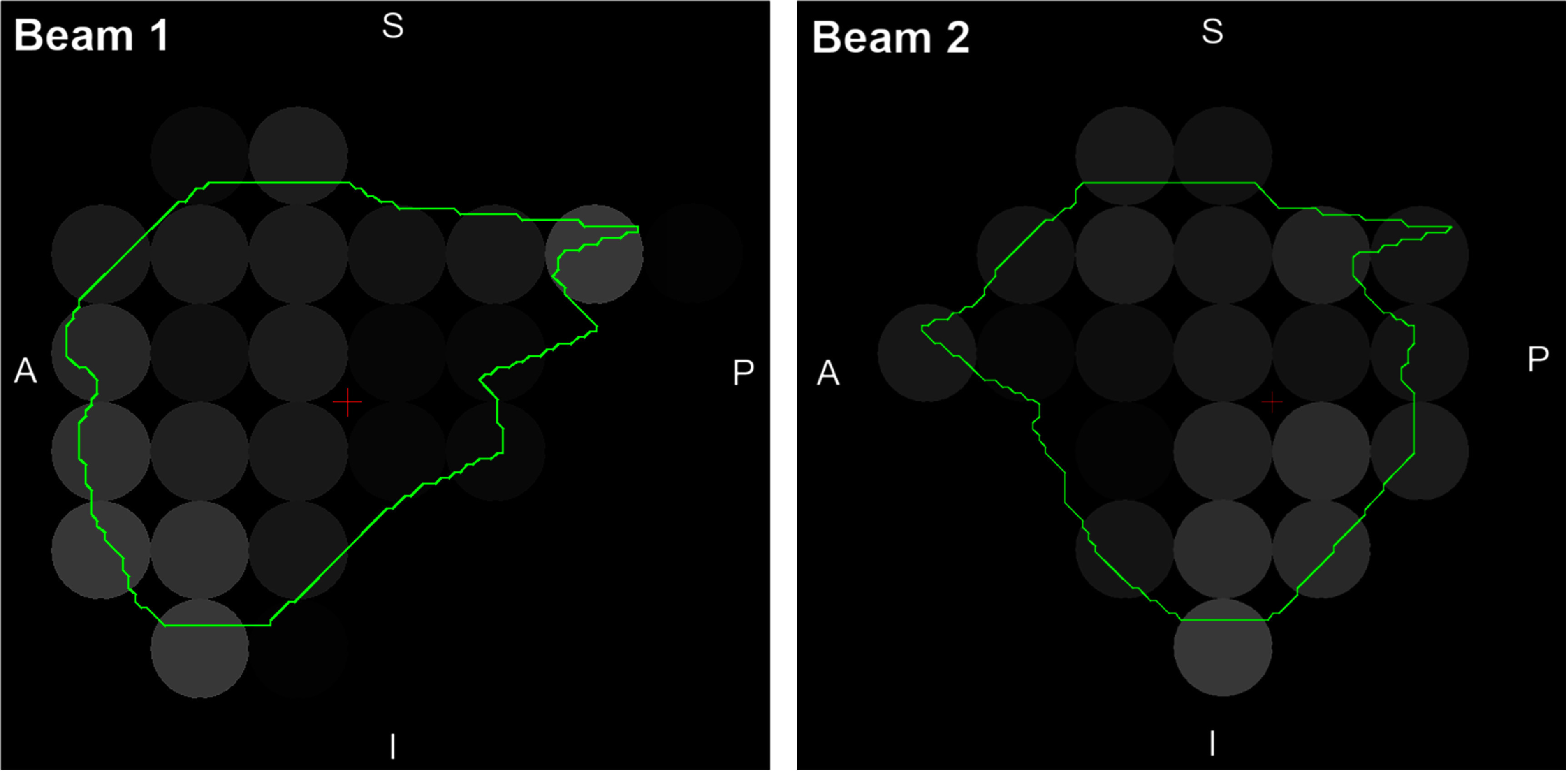
Beam’s eye views of the two beams in the electron plan with medium FLASH effect for patient 1. The green outline shows the GTV. The spots are shown with a nominal diameter of 4 mm, corresponding to the spot spacing, although the full width at half maximum is approximately twice this size. The greyscale of the spots indicates the monitor units delivered, lighter indicating greater intensity. Beam 1 is at gantry angle 75°, Beam 2 is at gantry angle 135° and couch angle is 0° for both beams (IEC 60601). A: Anterior, P: posterior, S: superior, I: inferior.

**Figure 4.**
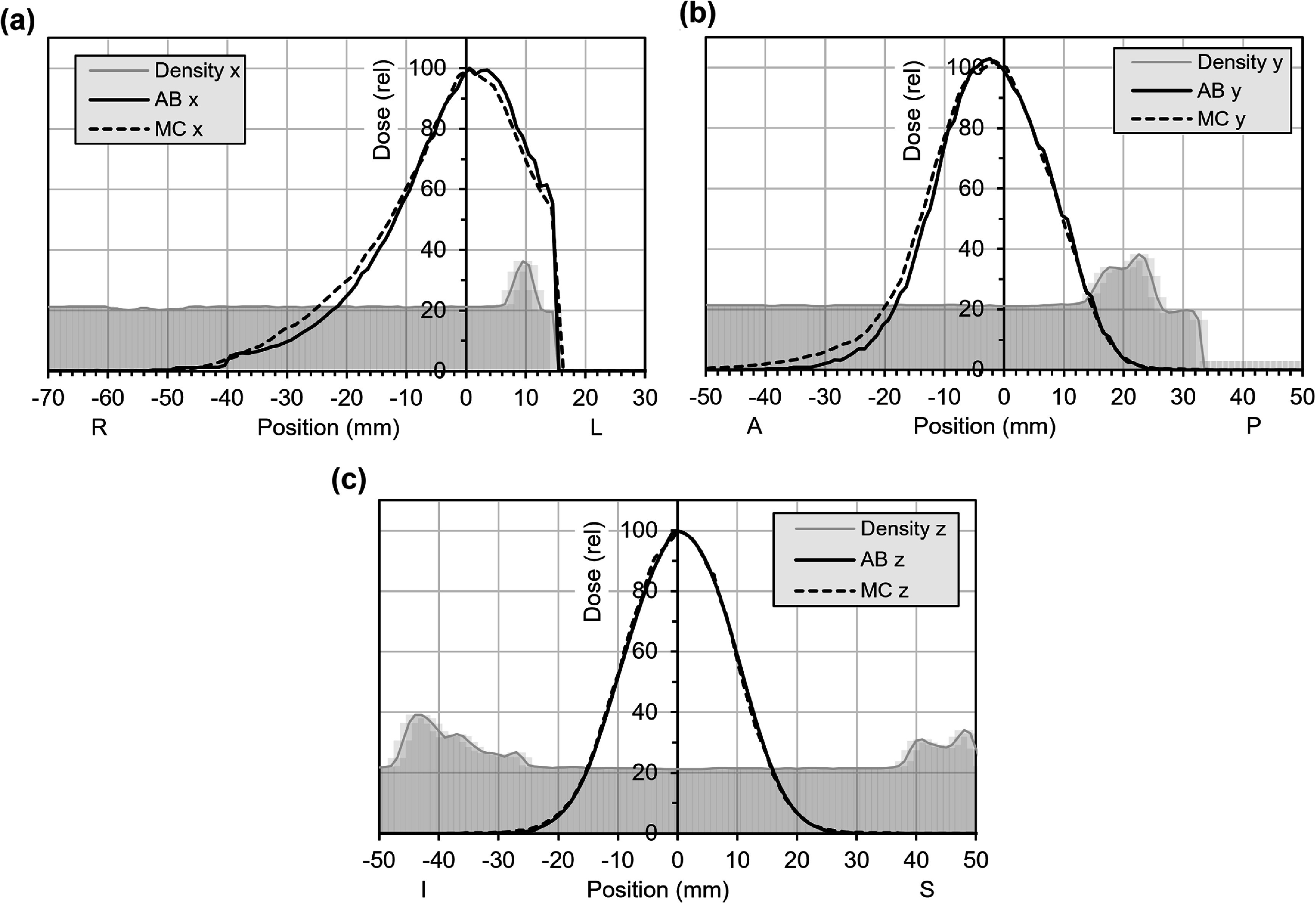
Dose profiles through the isocentre for the electron plan with medium FLASH effect in patient 1 (see figure 3). The dose is shown for the discrete ordinates solver in AutoBeam (AB, solid lines) and Monte Carlo simulation (MC, dotted lines) without FLASH RBE, normalised to the central axis. (a) Lateral profile, (b) antero-posterior profile, (c), supero-inferior profile. The greyscale background represents 20x the mass density of the patient, in g cm^−3^, i.e. 20 represents 1 g cm^−3^.

**Figure 5.**
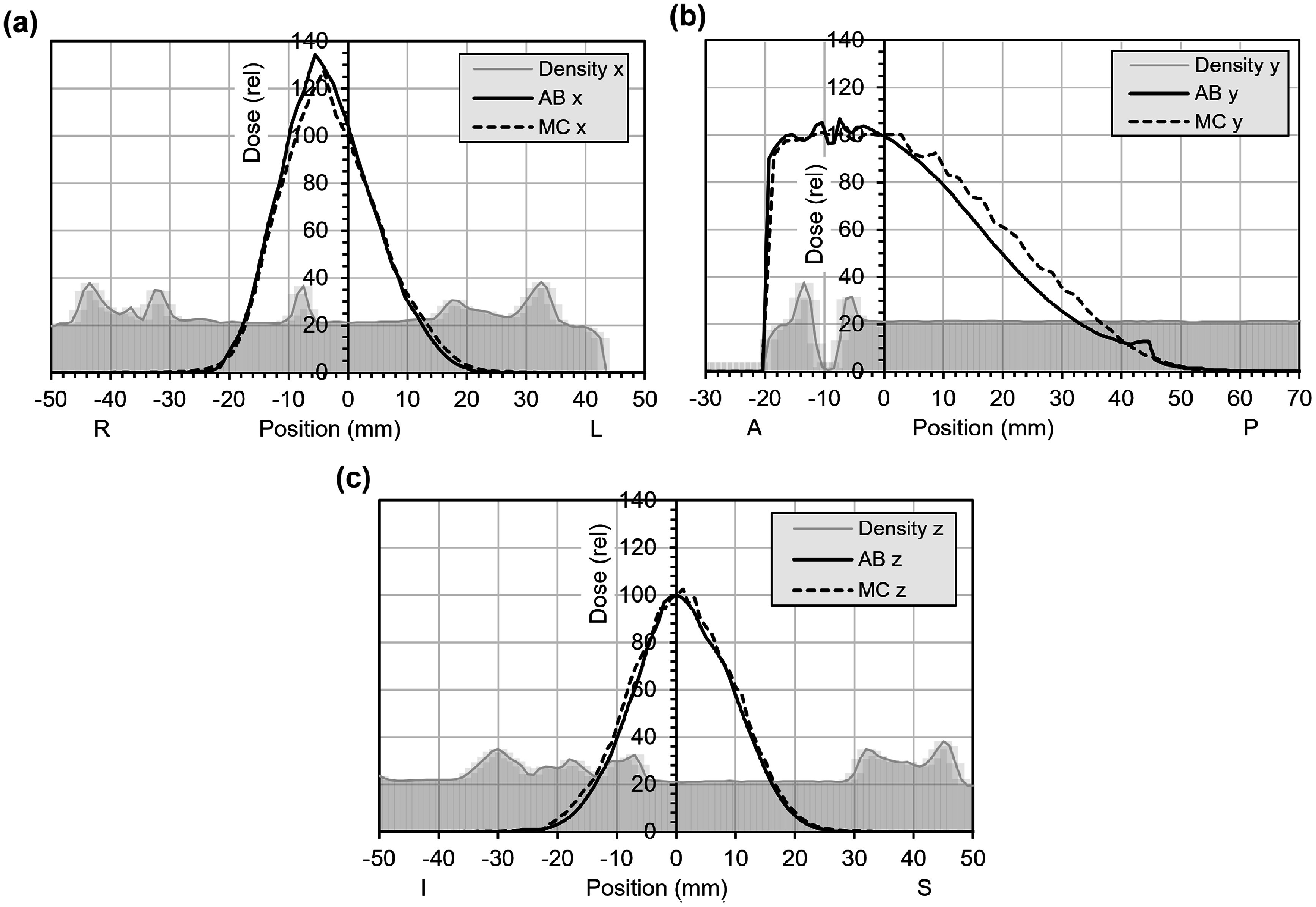
Dose profiles through the isocentre for the first beam of the electron plan with medium FLASH effect in patient 1 (see figure 3(a)), but shifted and rotated to irradiate through the frontal sinus. The dose is shown for the discrete ordinates solver in AutoBeam (AB, solid lines) and Monte Carlo simulation (MC, dotted lines) without FLASH RBE, normalised to the central axis. (a) Lateral profile, (b) antero-posterior profile, (c), supero-inferior profile. The greyscale background represents 20*x* the mass density of the patient, in g cm^−3^, i.e. 20 represents 1 g cm^−3^.

Typical spot patterns for the electron beam are shown in figure 3 for the case of medium FLASH effect. Figure 4 shows the results of the comparison with Monte Carlo simulation. Note that although medium FLASH effect is used to produce the plan, the dose comparison in figure 4 does not include RBE. There is good agreement on the profiles but less agreement along the lateral depth dose. Note, however that the profiles, although orthogonal to the patient, are oblique with respect to the beam axes, so the differences in dose fall-off are accentuated. Figure 5 shows a comparison with Monte Carlo simulation for more extreme heterogeneity. The beam calculated by discrete ordinates is slightly less penetrating than with Monte Carlo, although there is otherwise moderate agreement.

### 3.3. Patient study

**Figure 6.**
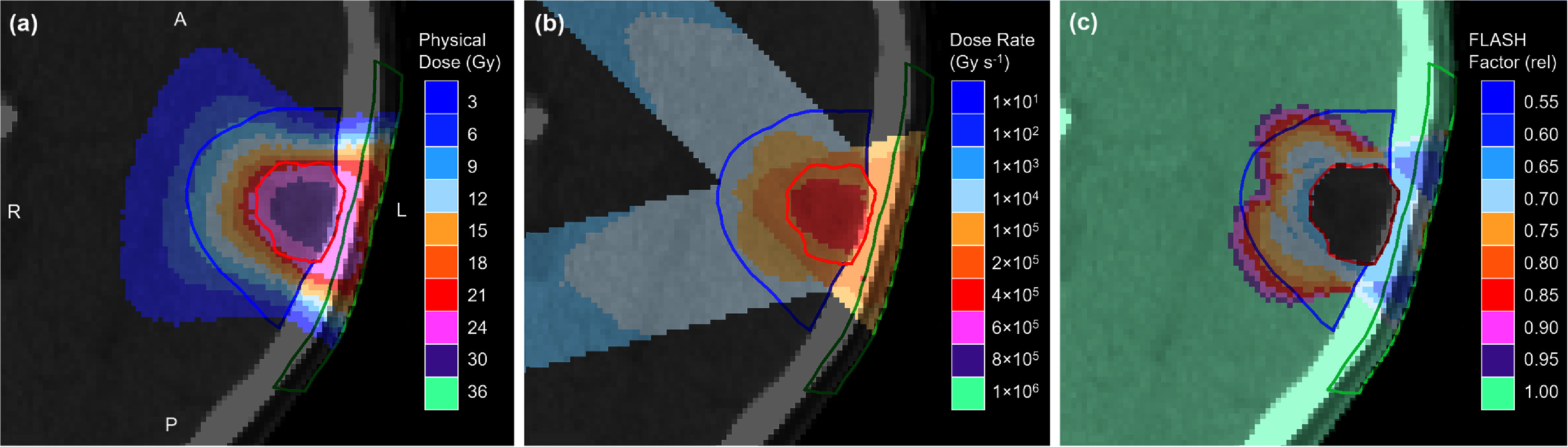
Transaxial distributions of (a) absorbed dose, (b) mean dose rate and (c) FLASH RBE from the electron plan with medium FLASH effect. The red contour is the GTV, the blue contour is proximal brain and the green contour is proximal skin. A: Anterior, P: posterior, L: left, R: right. Note the very extensive logarithmic scale on the dose rate.

The distributions of absorbed dose, mean dose rate and FLASH RBE are shown in figure 6 for the case of medium FLASH effect. The physical dose distributions are conformal, with maximum dose centred well on the GTV. Maximum skin dose is in the order of 27 Gy, which is close to the limit suggested by Timmerman (2022). The mean dose rate approximately follows the dose distribution, except that the distal region of the beam receives a noticeable dose rate, even though the dose itself is extremely low. The low dose in that region is a consequence of the classical fluence of equation (10), which calculates a small dose even at large depths, and although this is not intended to represent Bremsstrahlung, it serves that purpose in practice. The dose rate is then the quotient of this dose and time, so is also non-zero. Note, however, that the dose rate scale in figure 5 is exponential, so the actual dose rate in the distal region is relatively small. The FLASH RBE has a minimum, i.e. is most effective, at around 0.65 in the brain and around 0.60 in the skin. These are both areas where the effect is most needed.

**Figure 7.**
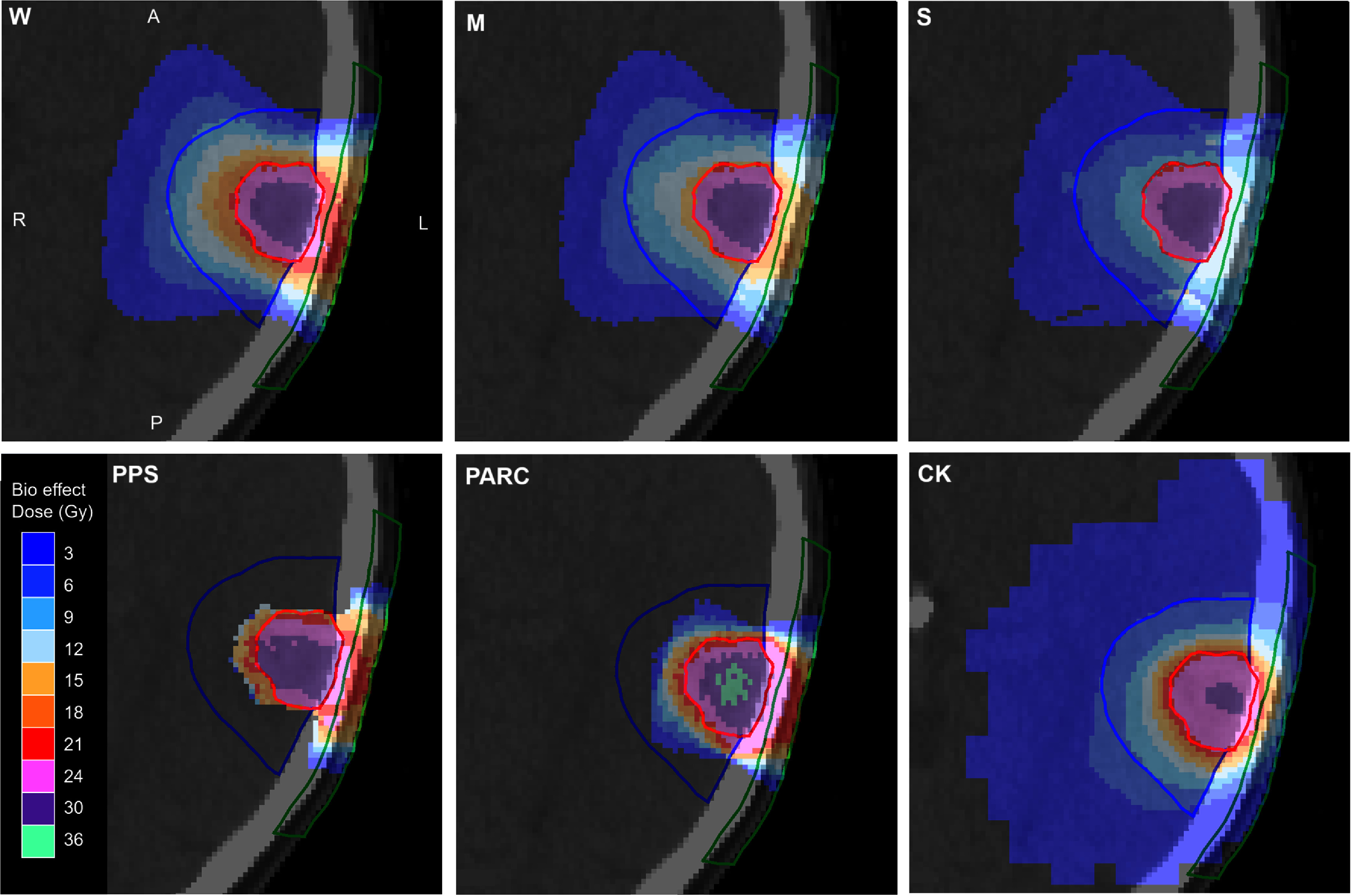
Transaxial dose distributions from electron, proton and photon plans including FLASH RBE (and proton RBE 1.1 where applicable). The red contour is the GTV, the blue contour is proximal brain and the green contour is proximal skin. W: electrons with weak FLASH effect, M: electrons with medium FLASH effect, S: electrons with strong FLASH effect, PPS: protons with passive scattering and medium FLASH effect, PARC: proton arc, CK: photons with Cyberknife. A: Anterior, P: posterior, L: left, R: right.

Figure 7 then shows how FLASH effect impacts on the total biologically effective dose, including FLASH effect, for both the electron plans and the comparison plans. As the FLASH effect becomes stronger, the proximal brain dose and the skin dose become lower, resulting in very conformal plans. The passively scattered proton plan is more conformal than electrons at low doses but is comparable to the electron plan with weak FLASH effect at higher doses. The proton arc plan is similar to the passively scattered proton plan but has a high skin dose. The Cyberknife photon plan is very conformal but has an extensive low-dose region due to the use of so many beams and also has a moderate skin dose.

**Figure 8.**
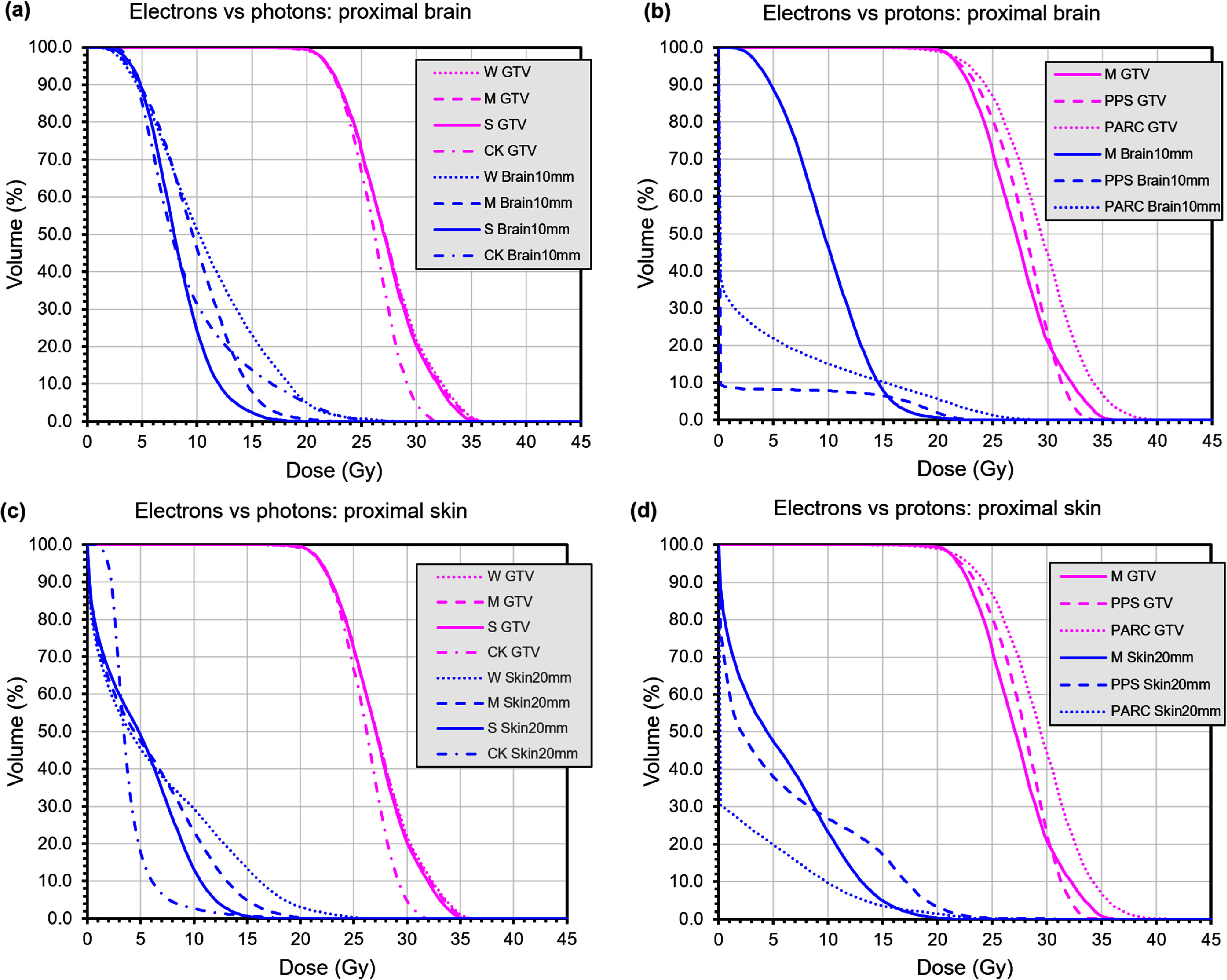
Dose-volume histograms of biologically effective dose including FLASH RBE (and proton RBE 1.1 where applicable) for GTV, proximal brain and proximal skin from electron, photon and proton plans. W: electrons with weak FLASH effect, M: electrons with medium FLASH effect, S: electrons with strong FLASH effect, CK photons with Cyberknife, PPS: protons with passive scattering and medium FLASH effect, PARC: proton arc.

Figure 8 shows mean dose-volume histograms for the six patients. Comparing electrons with photons, the GTV dose maximum is slightly higher with electrons but is well within the tolerance for this type of treatment of twice the prescribed dose, i.e. 42 Gy. Normal brain dose is favourable with electrons when FLASH effect is strong, particularly at the 12 Gy dose level. Skin dose is generally higher with electrons than with photons, but is limited successfully to the 27.5 Gy tolerance level.

Comparing electrons with protons, the electron plans give similar GTV dose to passively scattered protons, and a lower maximum dose than a proton arc, although the latter is still acceptable. Dose to normal brain is higher with electrons than either of the proton plans at the 12 Gy level, but maximum dose to skin is lower with electrons than with either of the proton plans. All of the plans are within accepted tolerance levels.

The key statistics for the plans are shown in table 5. Note that this table gives biologically effective dose, which includes RBE of 1.1 in the case of protons, and the FLASH modifying factor in the case of UHDR beams. The D_98%_ for GTV is set by prescription, so all of the plans have D_98%_ equal to 21.0 Gy. D_2%_ values for GTV are generally similar between plans although the Cyberknife plan has a lower value (*p* = 0.03). The lowest biologically effective V_12Gy_ for normal brain is from electrons with strong FLASH effect, since the high-dose rind around the GTV effectively disappears in this case, although this does not reach statistical significance (*p* = 0.2). A similar effect is observed with passively scattered protons to a lesser extent (*p* = 0.03). None of the plans irradiates skin to 25.5 Gy biologically effective dose, and this is confirmed by the D_0.035cm3_ statistics for skin. The lowest skin dose is seen with Cyberknife, due to the irradiation of the GTV from all directions rather than only from the proximal side, but the electron beam with strong FLASH effect is also favourable to skin. The strong FLASH effect is mainly the reason for this, as can be seen from figure 5, where the FLASH RBE is around 0.6.

**Table 5.** Median ± hemi-range dose statistics, based on relative biological effectiveness including FLASH effect, for electron, proton and photon plans.

	W^a^	M^a^	S^a^	PPS^a^	PARC^a^	CK^a^
GTV D_98%_ (Gy)	21.0 ± 0.0	21.0 ± 0.0	21.0 ± 0.0	21.0 ± 0.0	21.0 ± 0.0	21.0 ± 0.0
GTV D_2%_ (Gy)	**34.4 ± 2.9**	**34.3 ± 2.7**	**33.9 ± 2.9**	**31.9 ± 1.4**	**36.4 ± 2.4**	29.3 ± 2.2
Brain V_12 Gy_ (cm^3^)^b^	**6.4 ± 2.9**	4.2 ± 2.5	1.0 ± 2.3	**1.3 ± 0.8**	**2.4 ± 1.2**	3.6 ± 1.8
Skin V_25.5 Gy_ (cm^3^)	0.0 ± 0.1	0.0 ± 0.0	0.0 ± 0.0	0.0 ± 0.0	0.0 ± 0.0	0.0 ± 0.0
Skin D_0.035 cm3_ (Gy)	**22.2 ± 4.8**	17.7 ± 3.6	14.5 ± 1.5	**22.7 ± 2.7**	**16.4 ± 7.0**	8.3 ± 7.6
CI @ 100%^c^	1.70 ± 0.84	**1.02 ± 0.13**	**0.99 ± 0.03**	1.54 ± 0.13	**1.93 ± 0.28**	1.35 ± 0.26
CI @ 50%^c^	**12.68 ± 5.62**	**11.35 ± 4.88**	6.11 ± 3.06	5.86 ± 1.96	4.48 ± 0.89	4.83 ± 1.33

^a^ W: electrons with weak FLASH effect, M: electrons with medium FLASH effect, S: electrons with strong FLASH effect, PPS: protons with passive scattering, PARC proton arc, CK: photons with Cyberknife. Figures in bold are statistically significant at the 0.05 level with respect to CK.

^b^ Normal brain with GTV excluded.

^c^ CI: conformity index (volume of isodose/volume of GTV).

The conformity indices shown in table 5, which include the FLASH effect, are a useful summary of the different plans. At 100% dose level, i.e. 21 Gy, the Cyberknife plan has good conformality, i.e. a low conformity index, but the electron plans with medium and strong FLASH effect are almost completely conformal with conformity index of unity, due to the RBE reducing dose around the edge of the GTV (*p* < 0.05). At the 50% dose level, there is less difference between the plans, apart from the electron plans with weak and medium FLASH effect, which are less conformal (*p* = 0.03 with respect to Cyberknife). The electron plan with strong FLASH effect is similar to the other plans (*p* = 0.6). Figure 6 shows for medium FLASH effect that around the 50% level, i.e. approximately 10 Gy, the FLASH effect is still operative.

### 3.4. Uncertainties

Uncertainties in the study are estimated in table 6 by recalculating the electron plan for patient 1 using extreme parameters. The uncertainty in physical dose is limited to the ±5% simulated uncertainty, but the normal tissue and conformality statistics, which are affected by uncertainty in the FLASH parameters, vary rather more. However, none of the resulting statistics are clinically unreasonable.

**Table 6.** Uncertainties in the study, based on biologically effective dose with FLASH effect in patient 1.

	Lower estimate	Normal value	Upper estimate
GTV D_98%_ (Gy)	20.0	21.0	22.0
GTV D_2%_ (Gy)	32.8	34.2	35.6
Brain V_12 Gy_ (cm^3^)^a^	0.7	4.3	7.1
Skin V_25.5 Gy_ (cm^3^)	0.0	0.0	0.0
Skin D_0.035 cm3_ (Gy)	13.2	18.2	24.0
CI @ 100%^b^	0.96	0.99	1.70
CI @ 50%^b^	2.70	5.50	7.13

^a^ Normal brain with GTV excluded.

^b^ CI: conformity index (volume of isodose/volume of GTV), evaluated at 100% and 50% of 21 Gy.

## 4. Discussion

This study shows that a simple model of multiple electron scattering can be used as a fixed fluence in a discrete ordinates Boltzmann solver to calculate absorbed dose from a Gaussian beam. The Bolzmann solver is integrated into an inverse planning scheme so as to calculate dose at regular intervals during production of treatment plans. Use of a pulse-by-pulse dose rate model then allows estimation of RBE due to the FLASH effect in the UHDR of the beam. The results show that for superficial brain metastases, the electron beam is expected to reduce the irradiated volume of normal brain compared to current clinical practice of photon treatment using Cyberknife. Moreover, it is competitive with proton therapy with a single passively scattered FLASH beam or an arc at conventional dose rates. The skin dose with all of these techniques can be controlled to acceptable levels.

The use of the classical electron scattering model as a fixed fluence in the Boltzmann solver is valuable in the modelling of the PITZ beam. This is particularly due to the Gaussian nature of the beam itself, for which mathematical relations are available in ICRU 35. Further work is needed to implement cross sections for multiple scattering into the Boltzmann solver, so that the classical foundation is not needed. However, the discrete ordinates approach is most stable when there are fixed sources or a fixed fluence as the background, such as in photon transport where the unscattered exponential fluence can be used (Gifford *et al* 2006, Vassiliev *et al* 2010, Han *et al* 2013). Thus, some simple basis is likely to be needed.

Bremsstrahlung has been neglected in this work. Bremsstrahlung in a clinical electron beam mostly originates from the scattering foil and the beam collimation (Zhu *et al* 2001), neither of which is applicable in the present context of a scanned pencil beam. The study of Zhu *et al* (2001) calculates by Monte Carlo simulation of a 22 MeV beam of radius 50 mm that the bremsstrahlung due to electron interactions in the patient alone is less than 1.5% beyond the practical range of the beam. Likewise Sorcini *et al* (1996) calculate by analytical considerations of energy deposition and by Monte Carlo simulation that the maximum phantom bremsstrahlung component is located just within the continuous slowing down range, with a value for a 17.5 MeV beam of around 1.6%, lowering to less than 1% beyond the beam range. This radiation should therefore ideally be included but its absence in the present work is not expected to significantly affect the results.

The particular value of the discrete ordinates approach in the present study is in the area of efficiency and speed in the inverse planning context, but other methods such as Monte Carlo simulation are also useful (Amirkhanyan *et al* 2024, Gul and Şahmaran 2026). A simpler electron pencil beam model could also have been used for this work, as the dose calculation approach and the consequent modelling of UHDR treatment plans are largely separable. Inclusion of a multiple scattering model into the solver is the top priority, as this is beneficial for both photon and electron beams and may also be useful for proton beams. Addition of bremsstrahlung is then a further goal, to improve the accuracy of electron beams.

The results of the beam model are consistent with data measured using radiochromic film, but the film results themselves are subject to experimental uncertainty, particularly at the high dose rates used in the study. Karsch *et al* (2012) find no dose rate dependence of radiochromic film within a 5% statistical uncertainty at 1.5 × 10^10^ Gy s^−1^, and film has been used as a reference detector in comparisons with ionisation chambers, diode detectors and diamond detectors (Di Martino *et al* 2020). More recently, Jung *et al* (2025) show agreement of EBT-XD film with alanine to within 2% for measurements of average dose rate, instantaneous dose rate and dose per pulse at an instantaneous dose rate in the order of 3 × 10^5^ Gy s^−1^. However, a recent study by Del Sarto *et al* (2025) shows that in comparison to a FLASH diamond detector (PTW, Freiburg, Germany), EBT-XD film exhibits a dependence on average dose rate, instantaneous dose rate and dose per pulse of several percent at an instantaneous dose rate of 5 × 10^6^ Gy s^−1^. The film measurements in the present study are used in a relative context, and so are unlikely to be significantly affected by these effects, but the measurements should be taken as supporting the calculations rather than as definitive FLASH calibrations.

Several authors have reported on models for calculation of FLASH effect from distributions of dose and dose rate, particularly for proton therapy with pencil beam scanning (Folkerts *et al* 2020, Daartz *et al* 2024), and the present approach is similar to these. Recent work by Liu *et al* (2025) shows that both mean dose rate and dose per pulse affect the sparing of murine gastrointestinal tract, whereas Grilj *et al* (2025) find that mean dose rate is the most important factor, both studies using rectangular fields. In agreement with the latter work, the present study uses mean dose rate as the determining factor in the FLASH calculation. In the case of a scanned beam, the relationship between spatially localised instantaneous dose rate and mean dose rate is complex, but Sørensen *et al* (2022, 2024) make some progress in distinguishing between these parameters through *in vivo* experiments using rescanning of the pencil beam pattern and by using temporally separated pulses. The present study takes into account this knowledge and the treatment planning software is written in a generic form, so that only adjustment of tuning parameters is necessary to apply the methods to other electron accelerators. Although the exact width of the dose distribution depends on the pencil beam width and spot spacing, and the mean dose rate depends on the pulse structure of the accelerator that has been modelled, the results of the study are expected to be generally applicable. Investigation of a larger cohort of patients is necessary, and ultimately multi-centre clinical trials are needed to establish the true clinical impact.

Clinical delivery of stereotactic radiosurgery has traditionally been dominated by photon therapy, of which the Cyberknife plan is representative in this study. However, Atkins *et al* (2018) report on the use of 2–4 passively scattered proton beams in a large single-institution study of proton therapy, concluding that although resource-intensive, proton therapy is competitive with photon therapy. That study uses a median prescribed dose of 18 Gy RBE. The proton arc could eventually also be competitive in this field, particularly if used with spot reduction (van der Water *et al* 2020, Bertschi *et al* 2022, Fu *et al* 2024) and a single energy layer so as to avoid energy switching (Wang *et al* 2024).

The PHASER project (Maxim *et al* 2019) and other studies of electron accelerators (Krim etal 2025a, 2025b) have used fixed grids of apertures to provide conformality, but the electron beam at PITZ has a narrow width so it has been possible in this study to model the beam as a modulated set of pencil beams without additional collimation. This allows optimum use of the fast scanning system implemented on the beam line. Treatment of even smaller lesions than those considered is expected to be possible if the beam is focussed before scanning. In this case, the width of the beam can be reduced by about a factor of ten, allowing a very precise delivery of radiation. Treatment planning for this scenario would require careful consideration of beam separation and timing but the resulting dose distributions are expected to show high conformality. The Cyberknife beams in this study use multileaf collimation, which is flexible but not as conformal as cone apertures for the size of lesion being treated (Jang *et al* 2016).

A higher maximum beam energy is expected to increase the range of target depths that can be reached by the electron beam. Other studies show the value of increasing the energy to 250 MeV, into the range of very high energy electrons (VHEEs) (Bazalova-Carter *et al* 2015, Böhlen *et al* 2021, Stephan *et al* 2022, Bedford and Oelfke 2024). In that case, all brain metastases would be within reach of the beam. Muscato *et al* (2023) show that VHEE beams are comparable with other state-of-the-art photon and proton techniques for irradiation of brain tumours, with FLASH effect expected to produce further clinical benefits. Focussing the VHEE beam is also likely to produce improvements in conformality (Svendsen *et al* 2021). The most clinically accessible means of applying current technology to produce the benefits of UHDR is to use transmission scanning, as that enables the highest beam energy to be used, and this is shown by Gesualdi *et al* (2025) to be feasible with both electron and proton beams. Use of spatial fractionation in conjunction with VHEE and UHDR may also allow treatment with minimal side effects (Clements *et al* 2023). However, with all of these developments, there is a need to investigate treatment plans for large numbers of multiple metastases as these are increasingly encountered clinically.

Variation of parameters such as beam width and energy is a fruitful area of research. However, the value of the electron beam at present is largely due to its extremely high dose rate, so that the FLASH effect is expected to be stronger than with protons at UHDR. If the FLASH effect is found to be close to the strongest of the effects modelled in this study, the conformity index of the biologically effective dose is expected to be unity, so that no high-dose irradiation occurs outside of the GTV. Such a strong effect, together with the assumed biological model, remain to be verified practically and should not therefore be taken as general results. The FLASH RBE has been taken to be unity in the GTV itself, but in practice there may be some beneficial separation of tumour and normal tissue effects within this volume due to use of the UHDR. *In vivo* experiments using the beam in a laboratory setting should clarify just what the benefit of the dose rate is.

This study is an illustration of how radiotherapy with UHDR might perform. The actual clinical implementation of FLASH radiotherapy is a much larger task (Bourhis *et al* 2019b, Vozenin *et al* 2022), needing further research, dedicated beamlines and delivery systems that operate to appropriate safety standards, as well as accurate treatment planning and dosimetry. Initial implementation of UHDR electron therapy is likely to be with similar energy but much lower dose rates than those considered in this study (Giannini *et al* 2024). The outcome of these initial studies may then determine the trajectory towards increased energy and dose rate.

## Data Availability

The data cannot be made publicly available upon publication because they are not available in a format that is sufficiently accessible or reusable by other researchers. The data that support the findings of this study are available upon reasonable request from the authors.
